# The Gastric Juice

**Published:** 1834

**Authors:** 


### Art. VI.—The Gastric Juice.

*Experiments and Observations on the Gastric Juice, and the Physiology of Digestion.* By William Beaumont, M. D. Surgeon in the U. S. Army. Plattsburgh, 1833, pp. 280.

The public journals have already made our readers acquainted with the existence of Dr. Beaumont's book, in as much as the singular case of surgical disease, which remotely gave rise to it, has been made the subject of newspaper publication.

In the year 1822, when Dr. Beaumont was stationed at Mackinac, as a surgeon, a Canadian of the American Fur Company—Alexis St. Martin—accidentally received the contents of a musket loaded with duckshot, in his right side, when standing but three feet from the muzzle of the gun. The wound was terrible, and finally left a fistulous opening into his stomach, through which the Doctor was able at pleasure to draw off the contents of that organ. This extraordinary condition suggested and favored the experiments on the gastric juice and digestion, which make up the original parts of the work before us.

A lengthened introduction, setting forth the surgical history of the case, concludes with several graphical views of the orifice, seen under different states, and an account of the manner in which the gastric juice and the chymous contents of the stomach were extracted, which was as follows.

"*Mode of extracting the Gastric Juice.*—The usual method of extracting the gastric juice, for experiment, is by placing the subject on his right side, depressing the valve within the aperture, introducing a gum-elastic tube, of the size of a large quil, five or six inches into the stomach, and then turning him on the left side, until the orifice becomes dependent. In health, and when free from food, the stomach is *usually* entirely empty, and contracted upon itself. On introducing the tube, the fluid soon begins to flow, first by drops, then in an interrupted, and sometimes in a short continuous stream. Moving the tube about, up and down, or backwards and forwards, increases the discharge. The quantity of fluid ordinarily obtained is from four drachms to one and a half or two ounces, varying with the circumstances and condition of the stomach. Its extraction is generally attended by that peculiar sensation at the pit of the stomach, termed sinking, with some degree of faintness, which renders it necessary to stop the operation. The usual time of extracting the juice is early in the morning, before he has eaten, when the stomach is empty and clean.

On laying him horizontally on his back, pressing the hand upon the hepatic region, agitating a little, and at the same time turning him to the left side, bright yellow bile appears to flow freely through the pylorus, and passes out through the tube. Sometimes it is found mixed with the gastric juice, without this operation. This is, however, seldom the case, unless it has been excited by some other cause.

The chymous fluids are easily taken out by depressing the valve within the aperture, laying the hand over the lower part of the stomach, shaking a little, and pressing upwards. In this manner, any quantity necessary for examination and experiment can be obtained.

*Valve*.—The valve mentioned above, is formed by a slightly inverted portion of the inner coats of the stomach, fitted exactly to fill the aperture. Its principal and most external attachment is at the upper and posterior edge of the opening. Its free portions hangs pendulous, and fills the aperture when the stomach is full, and plays up and down, simultaneously with the respiratory muscles, when empty.

On pressing down the valve when the stomach is full, the contents flow out copiously. When the stomach is nearly empty, and quiescent, the interior of the cavity may be examined to the depth of five or six inches, if kept distended by artificial means; and the food and drinks may be seen entering it, if swallowed at this time, through the ring of the æsophagus. The perforation through the walls of the stomach, is about three inches to the left of the cardia, near the left superior termination of the great curvature. When entirely empty, the stomach contracts upon itself, and sometimes forces the valve through the orifice, together with an additional portion of the mucous membrane, which becomes completely inverted, and forms a tumour as large as a hen's egg. After lying on the left side, and sleeping a few hours, a still larger portion protrudes, and spreads out over the external integument, five or six inches in circumference, fairly exhibiting the natural rugæ, villous membrane, and mucous coat, lining the gastric cavity. This appearance is almost invariably exhibited in the morning before rising from his bed."—pp. 21–3.

We come, now, to what our author calls Preliminary Observations, which extend through 90 pages, and are divided into the following sections—*Aliment, Hunger and Thirst, Satisfaction and Satiety, Mastication, Insalivation and Deglutition, Digestion by the Gastric Juice, Appearance of the Villous Coat, and Motions of the Stomach, Chymification and Uses of the Bile and Pancreatic Juice.* Here, it will be perceived, is, in fact, the synopsis of a treatise on the Physiology of Digestion.

The first section, on Aliment, contains a number of sound observations, as for example,—that bulk is nearly as necessary in articles of diet as the nutrient principle; that all articles, whether vegetable or animal, are digested in the same manner, though with different degrees of rapidity; that the gastric juice is a solvent of every kind of nutritive matter, as caloric is of all bodies; and that different persons require very different quantities of food; under this head our author has given the results of a number of experiments on the comparative digestibility of different kinds of aliment. We shall extract the table entire.

"The following articles of the *materia alimentaria* have, in the course of these experiments, been submitted to the action of the stomach and the gastric fluids. I have attempted, in this table, to approximate towards a comparison of the digestibility of the several articles there mentioned. Precision, as to minutes, has not been attended to. When digestion has been accomplished two or three minutes either before or after a certain number of hours and quarters, I have set down the quarter to which it approached the nearest.

| Articles of Diet. | Mode of cook-ing. | Meal. | With b'rd or veg. or both. | | | Remarks. |
|---|---|---|---|---|---|---|
| | | | Exercise. | | Rest. | |
| | | | Mod. | Inc. | | |
| | | | h. m. | h. m. | h. m. | |
| Tripe, soused | fried | breakfast | 1 00 | | | |
| Pig's feet, do. | boiled | " | 1 00 | | | |
| Venison steak, fresh, | broiled | " | 1 35 | | | |
| Codfish, dry, | boiled | dinner | 2 00 | | | |
| Bread & Milk, | cold | " | 2 00 | | | |
| Turkey, | roast'd | " | 2 30 | | | |
| Goose, wild, | " | " | 2 30 | | | |
| Pig, young, | " | " | 2 30 | | | |
| Hash, meat & vegetables, | warm | breakfast | 2 30 | | | |
| Oysters, | raw | dinner | 2 45 | | | Oys. susp'd in stom. dur'g. exp't. |
| do. | stewed | dinner" | 3 30 | | | |
| do. | raw | breakfast | 3 00 | | | Nothing but a little dry bread or crackers taken at these meals. |
| do. | " | dinner | 3 00 | | | |
| do. | stewed | " | 3 30 | | | |
| Beef, fresh, fat and lean, | roast'd | " | 3 30 | | | |
| do. | " | " | 3 00 | | | |
| do. | " | breakfast | 2 45 | | | |
| do. | broiled | " | 3 00 | | | |
| do. | " | " | | | 3 45 | |
| do. | " | " | | 3 30 | | Exercised till fatigued. |
| do. | boiled | " | 4 00 | | | Morbid appearance of stomach. |
| do. | " | dinner | | 3 30 | | |
| do. | " | breakfast | 3 38 | | | Large proportion of fat. |
| do. | " | supper | | | 4 00 | Do. |
| do. | " | breakfast | | | 4 30 | Do. and in recumbent position. |
| do. | " | dinner | 3 30 | | | |
| do. | " | " | | | 4 00 | |
| do. | " | breakfast | | | 4 15 | |
| do. | " | " | 3 30 | | | |
| do. salted, | " | | | | 4 15 | |
| do. | " | dinner | 5 30 | | | |
| do. | " | " | 3 30 | | | |
| Pork, recently salted, | " | breakfast | 5 15 | | | |
| do. | " | " | 4 30 | | | |
| do. | " | " | 5 15 | | | Became angry during exp't. |
| do. | " | " | 6 00 | | | Unusually full meal. |
| do. | " | " | 4 30 | | | |
| do. | " | " | 4 30 | | | |
| do. | " | " | 4 30 | | | |
| do. | " | dinner | 4 30 | | | |
| do. | " | breakfast | | 4 00 | | |
| do. | " | dinner | | 3 30 | | |
| do. fresh, | roast'd | " | 6 30 | | | Unusually full meal. |
| do. steak, | broiled | " | 3 15 | | | |
| do. do. | " | breakfast | 4 30 | | | |
| Mutton, fat & lean, | roast'd | dinner | 3 15 | | | |
| do. | broiled | breakfast | | 3 00 | | |
| do. | " | " | 3 30 | | | |
| do. | " | " | 4 30 | | | Morbid appearance of stomach. |
| do. | " | dinner | 4 00 | | | |
| do. | " | breakfast | 4 30 | | | Full meal, coarsely masticated. |

| Articles of Diet. | Mode of cooking | Meal. | With br'd or veg. or both. Exercise. Mod. | Inc. | Rest. | Remarks. |
|---|---|---|---|---|---|---|
| | | | h. m. | h.m | h. m. | |
| Eggs, | h'd bld | breakfast | 3 30 | | | Bread or bread and coffee, no |
| do. | soft bld | " | 3 00 | | | veg. used with the eggs. |
| do. | h'd bld | dinner | 5 30 | | | Morbid appearance of stomach. |
| do. | soft bld | breakfast | 3 30 | | | Do. do. |
| do. | soft bld | dinner | 3 00 | | | |
| Sausage, | broiled | breakfast | 3 30 | | | With soft boiled eggs. |
| do. | " | dinner | 3 00 | | | |
| do. | fried | breakfast | 4 00 | | | Muslin bag containing same kind |
| do. | " | " | 5 00 | | | of diet, susp'd dur'g these expt's-- |
| do. | broiled | " | 3 30 | | | morbid appearance of stom. also. |
| do. | " | " | | 4 15 | | Full meal—severe exercise. |
| Fowls, (hens,) | boiled | " | 4 00 | | | With bread and coffee. |
| do. | " | dinner | 4 00 | | | With bread and water. |
| do. | " | " | 4 00 | | | do. do. |
| Veal, fresh, | broiled | breakfast | 4 00 | | | Muslin bag susp'd in stomach. |
| do. | " | dinner | 4 00 | | | |
| do. | " | breakfast | 4 00 | | | |
| do. | " | dinner | 4 45 | | | Morbid appearance of stomach. |
| do. | " | breakfast | | 3 45 | | |
| do. | " | dinner | 4 30 | | | |
| do. | " | breakfast | 5 30 | | | Morbid appearance of stomach. |

| | h. m. |
|---|---|
| Soup, made of fresh muscular fibre of beef and vegetables, | 4 00 |
| do. made of the hock, with vegetables, - - - | 4 15 |
| Bread, buttered, for breakfast, with coffee, [morbid appearance of stomach.] - - | 4 15 |
| do. buttered, for breakfast, with coffee, - - - | 3 45 |
| do. dry, for breakfast, with coffee, - - - | 4 00 |
| do. dry, for dinner, with dry mashed potatoes, - | 3 45 |

*Explanations to the abreviations in the preceding Table.*—br'd., for bread—veg., for vegetables—mod., for moderate—inc., for increased—susp'd., for suspended—dur'g., for during—exp't., for experiment—h'rd. bld., for hard boiled—h., for hour—m., for minute, &c. The figures denote the time of digestion, under the circumstances mentioned at the head of the column."—p. 40.

The author does not consider this table complete. The section from which it is extracted, concludes with the following paragraph:—

"The *quantity* of aliment is probably of more importance than the *quality*, to ensure health. The system requires much less than is generally supplied to it. The stomach disposes of a definite quantity. If more be taken than the actual wants of the economy require, the residue remains in the stomach, and becomes a source of irritation, and produces a consequent abberration of function, or passes in the lower bowels in an undigested state, and extends to them its deleterious influence. Dyspepsia is oftner the effect of over eating and over drinking than of any other cause."—p. 51.

Our own observations do not permit us to concur, *in extenso*, with our author in the opinion expressed in the close of this sentence. Over eating and drinking are often injurious to dyspeptics, but we must not confound this effect with the production of the disease. If a man has had his leg broken, it is injurious to the limb, for the weight of the body to be thrown upon it, though that weight did not occasion the fracture.

The second section, on Hunger and Thirst, must detain us but a moment. The author offers the following *Theory of Hunger:*—

"My impression is, that the sensation of hunger is produced by a *distention* of the gastric vessels, or that apparatus, whether vascular or glandular, which secrets the gastric juice; and is believed to be the effect of repletion by this fluid."—pp. 57–58.

For his reasonings in support of this *hypothesis*, we refer to the book itself, and, also, for his speculations on Thirst, and on Satisfaction and Satiety, which are the topics of the third section.

The fourth section, on Mastication, Insalivation and Deglutition, contains some judicious observations. The following remarks on the diffusion of food over the surface of the stomach, when introduced through the aperture of our patients stomach, are new and curious.

"The stomach will not admit of the introduction of food, even of a liquid kind, through the aperture, at a rapid rate. If a few spoonfuls of soup, or other liquid diet, be put in with a spoon or funnel, the rugæ gently closes upon it, and gradually diffuses it through the gastric cavity, entirely excluding more during this action. When a relaxation takes place, another quantity will be received in the same manner.

If the valvular portion of the stomach be depressed, and solid food be introduced, either in entire pieces, or finely divided quantities, the same gentle contraction, or grasping motion, takes place, and continues for fifty or eighty seconds; and will not allow of the introduction of another quantity until the above time has elapsed; when the valve may again be depressed, and more food be put in. Food and drinks will be received through the aperture no faster even when the stomach is entirely empty, than they are ordinarily received through the œsophagus.

When the subject of these experiments is so placed that the cardia can be seen and he be allowed to swallow a mouthful of food, the same contraction of the stomach, and closing upon the bolus, is invariably observed to take place at the œsophageal ring."—pp. 71–2.

The fifth section, on Digestion and the Gastric Juice, is one of considerable interest. The following conclusion, as to the length of time required for chymification, is, no doubt, true, as far as it regards the patient St. Martin:—

"The length of time consumed in the operation is various. It depends upon the quantity or quality of the ingestæ, the healthy or diseased state of the stomach, &c. In the various experiments which I have made, the medium time may be calculated at about three and a half hours."—p. 73.

The next section sets forth the results of Dr. Beaumont's experiments on the temperature of St. Martin's stomach, during digestion and exercise:—

"It has been suggested by many a physiologist, and positively asserted by some, that there is considerable increase of the temperature of the stomach during the digestion of a meal. But from the result of a great number of experiments and examinations, made with a view of ascertaining the truth of this opinion, in the empty and full state of the organ, and during different stages of chymification, I am convinced that there is no alteration of temperature, unless some other circumstance should produce it. Active exercise always elevates the temperature of the stomach, whether fasting or full, about one and a half degrees."—pp. 73–4.

We come now to the theories of Digestion. Our author adopts and confirms that of Spallanzani, which teaches that the stomach secretes a fluid which is the solvent of our food, and to which we have already referred. This fluid Dr. B. obtained, at pleasure, from the stomach of his patient, in the manner already pointed out.

In the year 1833, two distinguished professors of the University of Virginia, Emmett and Dunglison, analyzed a quantity of this fluid, and found it to contain, free *Muriatic and*

*Acetic acids*, *Phosphates and Muriates*, with bases of *Potassa, soda, magnesia*, and *lime*, and an *animal matter soluble in cold water but insoluble in hot.* The quantity of *muriatic acid* was so great as to astonish our experimenters. On the page following that in which he sets forth the above results, we came to an 'examination of the gastric fluid' by our learned and able friend, Professor Silliman of Yale College, but were disappointed in the results obtained. The principal of these was the existence of free *muriatic acid.*

We shall make no apology for the length of the following extract:—

"I think I am warranted, from the result of all the experiments, in saying, that the gastric juice, so far from being "inert as water," as some authors assert, is the most general solvent in nature, of alimentary matter—even the hardest bone cannot withstand its action. It is capable, *even out of the stomach* of effecting perfect digestion, with the aid of due and uniform degrees of heat, (100° Fahrenheit,) and gentle agitation, as will be seen in the following experiments.

The fact that alimentary matter is *transformed*, in the stomach, into chyme, is now pretty generally conceded. The peculiar process by which the change is effected, has been, by many, considered a problem in physiology. Without pretending to explain the exact *modus operandi* of the gastric fluid, yet I am impelled by the weight of evidence, afforded by the experiments, deductions and opinions of the ablest physiologist, but more by direct experiment, to conclude that the change effected by it on aliment is *purely chemical.* We must, I think, regard this fluid as a chemical agent, and its operation as a chemical action. It is certainly every way analogous to it; and I can see no more objection to accounting for the change effected on the food, on the supposition of a chemical process, than I do in accounting for the various and diversified modifications of matter, which are operated on in the same way. The decay of the dead bodies is a chemical operation, separating it into its elementary principles—and why not the solution of aliment in the stomach, and its ultimate assimilation into fibrine, gelantine and albumen? Matter, in a natural sense, is indestructible. It may be differently combined; and these combinations are chemical changes. It is well known that all organic bodies are composed of very few simple principles, or substances, modified by excess or diminution of some of their constituents.

The gastric juice appears to be secreted from numberless vessels, distinct and separate from the mucous follicles. These vessels, when examined with a microscope, appear in the shape of small lucid points, or very fine papillæ, situated in the intertices of the follicles. They discharge their fluid only when solicited to do so, by the presence of aliment, or by mechanical irritation.

Pure gastric juice, when taken directly out of the stomach of a healthy adult, unmixed with any other fluid, save a portion of the mucous of the stomach, with which it is most commonly, and perhaps always combined, is a clear, transparent fluid; inodorous; a little saltish; and very perceptibly acid. Its taste, when applied to the tongue, is similar to thin mucilaginous water, slightly asidulated with muriatic acid. It is readily diffusible in water, wine or spirits; slightly effervesces with alkalis; and is an effectual solvent of the *materia alimentaria.* It possesses the property of coagulating albumen, in an eminent degree; is powerfully antiseptic, checking the putrefaction of meat; and effectually restorative of healthy action, when applied to old, fœtid sores, and foul, ulcerating surfaces.

Saliva and mucus are sometimes abundantly mixed with the gastric juice. The mucous may be separated, by filtering the mixture through fine linen or muslin cambric. The gastric juice, and part of the saliva will pass through, while the mucus, and spumous or frothy part of the saliva, remains on the filter. When not separated by the filter, the mucus gives a ropiness to the fluid, that does not belong to the gastric juice, and soon falls to the bottom, in loose, white flocculi. Saliva imparts to the gastric juice, an azure tinge, and frothy appearance; and, when in large proportion, renders it fœtid in a few days; whereas the *pure* gastric juice will keep for many months, without becoming fœtid.

The gastric juice does not accumulate in the cavity of the stomach, until alimentary matter be received, and excite its vessels to discharge their contents, for the immediate purpose of digestion. It then begins to exude from its proper vessels, and increases in proportion to the quantity of aliment *naturally* required, and received. A definite proportion of aliment, only, can be perfectly digested in a given quantity of the fluid. From experiments on artificial digestion, it appears that the proportion of juice to the ingestæ, is greater than is generally supposed. Its action on food is indicative of its chemical character. Like other chemical agents, it *decomposes* or *dissolves*, and combines with, a fixed and definite quanity of matter, when its action ceases. When the juice becomes *saturated*, it refuses to dissolve more; and, if an excess of food have been taken, the residue remains in the stomach, or passes into the bowels, in a crude state, and frequently becomes a source of nervous irritation, pain and disease, for a long time; or until the *vis medicatrix naturæ* restores the vessels of this viscus to their natural and healthy action—either with or without the aid of medicine.

Such are the appearance and properties of the gastric juice; though it is not always to be obtained pure. It varies with the changing condition of the stomach. These variations, however, depend upon the admixture of other fluids, such as saliva, water, mucus, and sometimes bile, and, perhaps, pancreatic juice. The special solvent itself—the *gastric juice*—is, probably, invariably the same substance. Derangement of the digestive organs, slight febrile excitement, fright, or any sudden affection of the passions, cause material alterations in its appearance. Overburthening the stomach produces acidity and rancidity in this organ, and retards the solvent action of the gastric juice. General febrile irritation seems entirely to suspend its secretion into the gastric cavity; and renders the villous coat dry, red and irritable. Under such circumstances, it will not respond to the call of alimentary stimulus. Fear and anger check its secretion, also:—the latter causes an influx of bile into the stomach, which impairs its solvent properties.

When food is received into the stomach, the gastric vessels are excited by its stimulus to discharge their contents, when chymification commences. It has been a favorite opinion of authors, that food, after it has been received into the stomach, should "remain there a short period before it undergoes any change;"* the common estimate is one hour. But this is an erroneous conclusion, arising from inaccuracy of observation. Why should it remain there, unchanged? It has been received into the organ which is to effect an important change upon it —the gastric juice is ready to commence its work of solution soon after the first mouthful is swallowed; and, certainly, if we admit that the gastric juice performs the office of a chemical agent, which most physiologists allow, it is contrary to all our notions of chemical action, to allow it one moment to rest. It must commence its operation immediately. That it does so, is distinctly manifested by close observation of its action on food, in the healthy stomach."—pp. 83–7.

Dr. B. considers that moderate exercise conduces to digestion, by elevating, which in the case of St. Martin he ascertained it does, the temperature of the stomach about a degree and a half of Fahrenheit. The following paragraph appears to us to require elucidation:—

"Exercise, sufficient to produce moderate perspiration, increases the secretions from the gastric cavity, and produces an accumulation of a limpid fluid, within the stomach, slightly acid, and possessing the solvent properties of the gastric juice in an inferior degree. This is probably a mixed fluid, a small proportion of which is gastric juice."—p. 94.

Is the bile ever found in the stomach in a state of health? was formerly among the standing topics of debate in our medical societies. Dr. B. has made experiments and observations touching this matter, on his patient St. Martin, the results of which are set forth in the succeeding extract:—

"Bile is not essential to chymification. It is seldom found in the

*Paris On Diet, p. 39.

stomach, except under peculiar circumstances. I have observed that when the use of fat or oily food has been persevered in for some time, there is generally the presence of bile in the gastric fluids. Whether this be a pathological phenonenon, induced by the peculiarly indigestible nature of oily food; or whether it be a provision of nature, to assist the chymification of this particular kind of diet, I have not as yet satisfied myself. Oil is affected by the gastric juice with considerable difficulty. The alkaline properties of the bile may render it more susceptible of solution in this fluid, by altering its chemical character. Irritation of the pyloric extremity of the stomach with the end of the elastic tube, or the bulb of the thermometer, generally occasions a flow of bile into this organ. External agitation, by kneading with the hand, on the right side, over the regions of the liver and pylorus, produces the same effect. It may be laid down as a general rule, however, subject to the exceptions above mentioned, that bile is not necessary to the chymification of food in the stomach. MAGENDIE says, "I believe that in certain morbid conditions, the bile is not introduced into this organ," (the stomach;) inferring, that in a healthy state, it is always to be found there. There can hardly be a greater mistake. With the exceptions that I have mentioned, it is never found in the gastric cavity, in a state of health; and it is only in "certain morbid conditions" that it is found there."—p. 95.

The following paragraphs are deserving of attention:—

"The progress of chyme from the stomach is gradual. Portions of chyme, as they become formed, pass out, and are succeeded by other portions. In the early stages, the passage of chyme into the duodenum, is more slowly effected than in the later stages. At first, it is more mixed with the undigested portions of aliment, and is probably separated with difficulty, by the powers of the stomach. In the later stages, as the whole mass becomes more chymified, and fitted for the translation, the process is more rapid; and is accelerated by a peculiar contraction of the stomach, a description of which will be found in the next section. It appears to be a provision of nature, that the chyme, towards the latter stages of its formation, should become more stimulating, and operate on the pyloric extremity of the stomach, so as to produce this peculiar contraction.

After the expulsion of the last particles of chyme, the stomach becomes quiescent, and no more juice is secreted, until a fresh supply of food is presented for its action, or some other mechanical irritation is applied to its internal coat.

Water and alcohol are not affected by the gastric juice. Fluids, of all kinds, are subject to the same exemption, unless they hold in solution or suspension some animal or vegetable aliment. Fluids pass from the stomach very soon after they are received, either by absorption, or through the pylorus."—pp. 96–7.

The section on the appearance of the inner coat of the stomach, as seen in St. Martin, and the influence of different kinds of irritation on the secretion of the gastric juice, is among the most interesting portions of our author's book.

"The inner coat of the stomach, in its natural and healthy state, is of a light, or pale pink colour, varying in its hues, according to its full or empty state. It is of a soft, or velvet-like appearance, and is constantly covered with a very thin, transparent, viscid mucous, lining the whole interior of the organ.

Immediately beneath the mucous coat, and apparently incorporated with the villous membrane, appear small, spheroidal, or oval shaped, glandular bodies, from which the mucous fluid appears to be secreted.

By applying aliment, or other irritants, to the internal coat of the stomach, and observing the effect through a magnifying glass, innumerable minute lucid points, and very fine nervous or vascular papillæ, can be seen arising from the villous membrane, and protruding through the mucous coat, from which distills a pure, limpid, colourless, slightly viscid fluid. The *fluid,* thus excited, is invariably distinctly acid. The *mucous* of the stomach is less fluid, more viscid or albuminous, semi-opaque, sometimes a little saltish, and does not possess the slightest character of acidity. On applying the tongue to the mucous coat of the stomach, in its empty, unirritated state, no acid taste can be perceived. When food, or other irritants, have been applied to the villous membrane, and the gastric papillæ excited, the acid taste is immediately perceptible. These papillæ, I am convinced, from observation, form a part of what is called by authors, the villi of the stomach. Other vessels, perhaps absorbing as well as secretory, compose the remainder. That some portion of the villi form the excretory ducts of the vessels, or glands, I have not the least doubt, from innumerable, ocular examinations of the process of secretion of gastric juice. The invariable effect of applying aliment to the internal, but exposed part of the gastric membrane, when in a healthy condition, has been the exudation of the solvent fluid, from the above mentioned papillæ.—Though the *apertures* of these vessels could not be seen, even with the assistance of the best microscopes that could be obtained; yet the points from which the fluid issued was clearly indicated by the gradual appearance of innumerable, very fine, lucid specks, rising through the transparent mucous coat, and seeming to burst, and discharge themselves upon the very points of the papillæ, diffusing a limid, thin fluid over the whole interior gastric surface. This appearance is conspicuous only during alimentation, or chymification. These lucid points, I have no doubt, are the termination of the excretory ducts of the gastric vessels or glands, though the closest and most accurate observation may never be able to discern their distinct apertures.

The fluid, so discharged, is absorbed by the aliment in contact, or collects in small drops, and trickles down the sides of the stomach, to the more depending parts, and there mingles with the food, or whatever else may be contained in the gastric cavity. This fluid, the efficient cause of digestion—the true gastric juice of Spallanzani, I have no doubt—has generally been obtained, for experiment, by mechanical irritation of the internal coat of the stomach, produced by the introduction of a gum-elastic tube, through which it has been procured.

The gastric juice never appears to be accumulated in the cavity of the stomach while fasting; and is seldom, if ever, discharged from its proper secerning vessels, except when excited by the natural stimulus of aliment, mechanical irritation of tubes, or other excitants. When aliment is received, the juice is given out in exact proportion to its requirements for solution, except when more food has been taken than is necessary for the wants of the system.

When mechanical irritation by a non-digestible substance, as the elastic tube, stem of the thermometer, &c. has been used, the secretion is probably less than when the irritation has been produced by such substances, as are readily dissolved in the gastric juice. Alimentary stimulus, when taken into the stomach, is diffused over the whole villous surface, and excites the gastric vessels, generally, to excrete their fluids copiously; whereas the irritation of tubes, &c. is local, and produces only a partial excitement of the vessels, and a scanty flow of the gastric juice. Hence, the slowness in obtaining the clear fluid from the empty stomach, through the tube. I have never, on numerous trials, been able to obtain, at any one time, more than one and a half, or two ounces of this fluid, after the stomach had disposed of its alimentary matters, however long the period of abstinence had been. The discharge of this small quantity has generally been excited by the introduction of the tube. Ten, fifteen, or more minutes, were necessary to collect even this small quantity. Whenever fluid was obtained in larger quantity, as was sometimes the case, it invariably contained more than the usual quantity of mucus.

On viewing the interior of the stomach, the peculiar formation of the inner coats are distinctly exhibited. When empty, the rugæ appear irregularly folded upon each other, almost in a quiesent state, of a pale pink colour, with the surface merely lubricated with mucus. On the application of aliment, the action of the vessels is increased; the colour brightened; and the vermicular motions excited. The small gastric papillæ begin to discharge a clear, transparent fluid, (the alimentary solvent,) which continues abundantly to accumulate, as aliment is received for digestion.

If the mucous covering of the villous coat be wiped off, with a sponge or handkerchief, during the period of chymification, the membrane appears roughish, of deep pink colour at first; but in a few seconds, the follicles and fine papillæ begin to pour out their respective fluids, which, being diffused over the parts abraded of mucus, restore to them their peculiar soft and velvet-like coat, and pale pink colour, corresponding with the undisturbed portions of the membrane; and the gastric juice goes on accumulating, and trickles down the sides of the stomach again.

If the membrane be wiped off when the stomach is empty, or during the period of fasting, a similar roughness, and deepened colour appear, though in a less degree; and the mucous exudation is more slowly restored. The follicles appear to swell more gradually. The fluids do not accumulate in quantity sufficient to trickle down, as during the time of chymification. The mucous coat only, appears to be restored.

The foregoing, I believe to be the natural appearances of the internal coat of the stomach, in a healthy condition of the system."—pp. 103—7.

On the peristaltic motions of the stomach, we have the following observations:

"These contractions and relaxations of the muscular fasciculi, do not observe any very *exact* mode. Their motions are modified by various circumstances, such as the stimulant or non-stimulant property of the ingestæ, the healthy or unhealthy state of the internal coat of the stomach; by exercise, and by repose, &c., &c.

The ordinary course and direction of the revolutions of the food, are first, after passing the œsophageal ring, from right to left, along the small arch; thence, through the large curvature, from left to right. The bolus, as it enters the cardia, turns to the left; passes the aperture; descends into the splenic extremity; and follows the great curvature towards the pyloric end. It then returns, in the course of the smaller curvature, makes its appearance again at the aperture, in its descent into the great curvature, to perform similar revolutions.

Such I have ascertained to be the revolutions of the contents of the stomach, from being able to identify particular portions of food, and from the fact, that the bulb of the thermometer, which has been frequently introduced during chymification, invariably indicates the same movements. These revolutions are completed in from one to three minutes. They are probably induced, in a great measure, by the circular or transverse muscles of the stomach, as indicated by the spiral motion of the thermometer, both in descending to the pyloric portion, and ascending to the splenic.† These motions are slower at first than after chymification has considerably advanced.

While these revolutions of the contents of the stomach are progressing, the trituration or agitation is also going on. There is a perfect admixture of the whole ingestæ, during the period of alimentation and chymification. There is nothing of the distinct lines of separation between old and new food, and peculiar central or peripheral situation of crude, as distinguished from chymified aliment, said to have been observed by PHILIP, MAGENDIE and others, in their experiments on

† The terms "descending" and "ascending," are used here, as well as in many other places, relatively; because the examinations were generally made while the man was lying on his right side.

dogs and rabbits, to be seen in the human stomach; at least in that of the subject of these experiments. The whole contents of the stomach, until chymification be nearly complete, exhibit a heterogenous mass of solids and fluids; hard and soft; course and fine; crude and chymified; all intimately mixed, and circulating promiscuously through the gastric cavity, like the mixed contents of a closed vessel, gently agitated, or turned in the hand.

If a mouthful of some tenacious food be swallowed, after digestion is considerably advanced, it will be seen passing the opening, to the great curvature; and in the course of about one and a half or two miutes, it will re-appear, with the general circulating contents, more or less broken to pieces, or divided into smaller pieces; and very soon loses its identity. This agitating motion has the effect, and is undoubtedly designed, to break up the bolus, as well as to separate the external and chymified portion of the particles of food, and allow the undigested portions to come in contact with the gastric juice' the proper solvent. If the motions were simply revolutionary, the central portions would retain their situation, until the outer, or chymified part, had passed into the duodeum, in successive parcels; which, it is evident, would very much retard the process of digestion.

As the food becomes more and more changed from its crude to its chymified state, the accidity of the gastric fluids is considerably increased; more so in vegetable than animal diet; and the general contractile force of the muscles of the stomach is augmented in every direction; giving the contained fluids an impulse towards the pylorus.

It is probable, that from the very commencement of chymification —from the time that food is received into the stomach—until that organ becomes empty, portions of chyme are constantly passing into the duodenum, through the pyloric orifice, as the mass is presented at each successive revolution. I infer this, from the fact that the volume is constantly decreasing. This decrease of volume, however, is slow at first; but is rapidly accelerated towards the conclusion of digestion, when the whole mass becomes more or less chymified. This accelerated expulsion appears to be effected by a peculiar action of the transverse muscles, or rather of the *transverse band*, as described by Spallanzani, Haller, Cooper, Sir E. Home, and others, in their experiments on animals. This band is situated near the commencement of the more conically shaped part of the pyloric extremity, three or four inches from the smaller end. In attempting to pass a long glass thermometer tube, through the aperture, into the pyloric portion of the stomach, during the latter stages of digestion, a forcible contraction is first perceived at this point, and the bulb is stopped. In a short time, there is a gentle relaxation, when the bulb passes without difficulty, and appears to be drawn, quite forcibly, for three or four inches, towards the pyloric end. It is then released, and forced back, or suffered to rise again; at the same time, giving to the tube a circular, or rather spiral motion, and frequently revolving it completely over. These motions are distinctly indicated, and strongly felt, in holding the end of the tube between the thumb and finger; and it requires a pretty forcible grasp to prevent it from slipping from the hand, and being drawn suddenly down to the pyloric extremity. When the tube is left to its own direction, at these periods of contraction, it is drawn in, nearly its whole length, to the depth of ten inches: and when drawn back, requires considerable force, and gives to the fingers the sensation of a strong *suction* power, like drawing the piston from an exhausted tube. This ceases as soon as the relaxation occurs, and the tube rises again, of its own accord, three or four inches, when the bulb seems to be obstructed from rising further; but if pulled up an inch or two, through the stricture, it moves freely in all directions in the cardiac portions, and mostly inclines to the splenic extremity, though not disposed to make its exit at the aperture.

Above the contracting band, and towards the splenic portion of the stomach, the suction or grasping motion is not perceptible; but when the bulb is pushed down to this point, it is distinctly felt to be grasped, and confined in its movements.

These peculiar motions and contractions continue until the stomach is perfectly empty, and not a particle of food or chyme remains; when all becomes quiescent again.

If the bulb of the thermometer be suffered to be drawn down to the pyloric extremity, and detained there for a short time, or if the experiment be repeated too frequently, it causes severe distress, and a sensation like cramp, or spasm, which ceases on withdrawing the tube; but leaves a sense of soreness and tenderness at the pit of the stomach.

These peculiar contractions and relaxations, mentioned above, succeed each other, at irregular intervals, of from two to four or five minutes. Simultaneously with the contractions, there is a general shortening of the fibres of the stomach. This organ contracts upon itself in every direction; and its contents are compressed with much force. The valvular portion of the stomach is firmly thrust into the aperture; closing the orifice; preventing the egress of aliment; and obstructing the view of the interior. During the intervals of relaxation, the rugæ perform their vermicular actions, the undulatory motions of the fluids continue, and the alimentary and chymous mass appear, revolving as before, promiscuously mixed, through the splenic and cardiac portions.

All these facts, taken together, will, I think, rationally admit of the following explanation. The longitudinal muscles of the whole stomach, with the assistance of the transverse ones of the splenic and central portions, carry the contents into the pyloric extremity. The circular or transverse muscles contract progressively, from left to right. When the impulse arrives at the *transverse band*, this is excited to a more forcible contraction, and, closing upon the alimentary matter and fluids contained in the pyloric end, prevents their regurgitation. The muscles of the pyloric end, now contracting upon the contents detained there, separate and expel some portions of the chyme. It appears that the crude food excites the contractile power of the pylorus, so as to prevent its passage into the duodenum, while the thinner, chymified portion is pressed through the valve, into the intestine. After the contractile impulse is carried to the pyloric extremity, the circular band, and all the transverse muscles, become relaxed, and a contraction commences in a reversed direction, from right to left, and carries the contents again to the splenic extremity, to undergo similar revolutions.

It would appear, then, that the discharge of the chyme from the stomach, is effected by *mechanical* impulse. But, I confess, I do not like to give an opinion. I state the circumstances as they have occurred. The idea of mechanical force, I admit, is liable to objection; but, perhaps, not more so than that of the *selecting* power of the pylorus. Whatever bias I may have in favour of the former method, has been forced upon me by the deductions of experiment and observation."—pp. 110—116.

The last section of the preliminary observations, on Chylification, need not detain us, as our great object is to present what is new in Dr. Beaumont's book. The aperture in St. Martin's *stomach*, would of course afford but little aid in an inquiry into the functions of the *bowels, liver and pancreas*. It is true that our author attempted to form chyle out of the body by the addition of bile, muriatic acid and pancreatic juice, but with no striking result. With these remarks we conclude our account of the first part of the work before us, and now proceed to a notice of the *Experiments and Observations*.

We shall not attempt to follow our author through the narrative of his several series of experiments, the first of which was performed in 1825, the last in 1833. We have stated most of the results obtained by him, in our copious extracts from his preliminary observations. The question whether the gastric juice is secreted by the stimulus of food, very properly attracted Dr. B's attention, and as it is one of interest, we shall extract entire the experiments which relate to it.

"To ascertain whether the Gastric Juice be *accumulated* in the stomach, during periods of fasting, or even from the immediate and direct influence of hunger, I made the following experiments.

*Experiment* 9.—*Dec*. 5, 1829.—At 8 o'clock, A. M., after twelve hours abstinence from either food or drinks, I introduced, at the perforation, a gum-elastic tube, and drew off a drachm or two only of the gastric juice.—There was no accumulation in the stomach.

*Experiment* 10.—*Dec*. 12.—At 3 o'clock, P. M., introduced tube—

could procure two or three drachms only—this was secreted on the irritation of the tube. Stomach contained none in a free state.

*Experiment* 11.—*Dec.* 14.—At 10 o'clock, P. M., after eighteen hours fasting, introduced tube, and drew off one and a half ounces of gastric juice. It was clear, and almost transparent; tasted a little saltish and acid, when applied to the tongue, similar to thin mucilage of gum arabic, slightly acidulated with muriatic acid. There was no accumulation in the stomach when the tube was introduced.

*Experiment* 12.—*March* 13, 1830.—At 10 o'clock, A. M.—stomach empty—introduced tube; but was unable to obtain any gastric juice. On the application of a few crumbs of bread to the inner surface of the stomach, the juice began slowly to accumulate, and flow through the tube. The crumbs of bread adhered to the mucous coat, soon became soft, and began to dissolve and digest. On viewing the villous membrane *before* applying the bread crumbs, the mucous coat and subjacent follicles only, could be observed; but immediately *afterwards*, small, sharp papillæ, and minute lucid points, situated in the interstices of, and less than, the mucous follicles, became visible; from which exuded a clear, transparent liquor. It then began to run through the tube.

*Experiment* 13.—*March* 18.—At 6 o'clock, P. M., after fasting from 8 o'clock, A. M., introduced tube—obtained one and a half ounces gastric juice, after having kept up the irritation, by moving the tube from point to point, for twelve or fifteen minutes. No accumulation of free juice in the stomach.

*Experiment* 14.—*Jan.* 26, 1831.—At 9 o'clock, A. M.—stomach empty—extracted one ounce gastric, slowly through the tube, with the usual admixture of mucus. Introduced food, and it began directly to flow more freely through the tube.

*Experiment.* 15—*Jan.* 27.—At 8 o'clock, A. M.—stomach empty—introduced elastic tube, and obtained one and a half drachms of gastric juice, by very slow distillation. Applied crumbs of bread to the villous coat, and the juice began immediately to flow through the tube.

*Experiment* 16·—*March* 6.—At 8 o'clock, A. M., extracted two ounces gastric juice, and added it to two ounces of *Madeira wine.* No visible change was produced—no coagulæ formed. They united, like pure water and wine. Heat produced no other effect.

*Experiment* 17.—*March* 7.—At 6 o'clock, P. M.—stomach empty—extracted one and a half ounces of juice, and mixed it with the same quantity of *Jamaica spirits.* Effect same as with wine.

*Experiment* 18.—*March* 8.—At 8 o'clock, A. M.—stomach empty—extracted one and a half ounces of gastric juice.

*Experiment* 19.—*March* 12.—At 9 o'clock, A. M.—stomach empty

—extracted one and a half ounces of gastric juice. Put this in a bottle.

*Experiment* 20.—*March* 13.—At 11 o'clock, A. M.—stomach empty—extracted two ounces of juice.

*Experiment* 21.—*March* 14.—At 12 o'clock, M.—stomach empty—extracted two ounces of juice.

*Experiment* 22.—*March* 15.—At 4 o'clock, P. M.—stomach empty—extracted one and a half ounces gastric juice.

*Experiment* 23.—*March* 16.—At 5 o'clock, P. M., introduced tube—could obtain no clear gastric juice. A little acrid fluid and frothy mucus, only, could be extracted. Villous membrane red and dry. St. Martin complained of some headache, pain and distress about the scrobiculus cordis, lassitude and loss of appetite. Directed him to take half an ounce of *tincture of aloes and myrrh*, at 9 o'clock, P. M. This moved his bowels several times next morning. Little or no change was apparent in the appearance of the inner coat of the stomach; if any, it was a little more moist, and a shade paler, after the operation of the tincture. Gastric juice could again be obtained, but in less than usual quantity.

It would seem, from the preceding experiments, that the stomach contains *no gastric juice*, in a free state, when aliment is not present. Any digestible or irritating substance, when applied to the internal coat, excites the action of the gastric vessels. Hence, I infer that the fluid, in these experiments, was incited to discharge itself by the irritation of the tube used in extracting it.

If, as is contended for by some, a *part* of the fluid be discharged into the stomach during a fast, I see no reason why nature should withhold the other part. If we may be allowed to argue, independent of more certain data, one great objection to the opinion that the stomach contains gastric juice, in a free state, when food is witholden from it, exists in the danger of its passing out through the pyloric orifice; and thus depriving the succeeding meal of the benefit of its solvent action. It is probable that the pyloric orifice opposes no resistance to its egress; but is obedient to its summons. In this way we may account for its admitting chyme, which is an admixture, or rather, combination, of gastric juice and food, to obey the expulsive motions of the stomach, and pass out. They both appear to excite the peculiar contraction of the pyloric end of the stomach, mentioned in a former part of this work. Besides, there would be danger of the gastric juice being weakened, by the introduction of large quantities of water, or other fluids, in the intervals of eating, and thus lose its energy, and concentrated solvent properties.

The last experiment has considerable pathological importance. In febrile diathesis, very little or no gastric juice is secreted. Hence, the importance of withholding food from the stomach in febrile complaints. It can afford no nourishment; but is actually a source of irritation to that organ, and, consequently, to the whole system. No solvent can be secreted under these circumstances; and food is as insoluble in the stomach, as lead would be under ordinary circumstances."—pp. 131—38.

We may add that on a variety of occasions, Dr. B. perceived that irritation of the mucous membrane of the stomach, was followed by an immediate secretion.

From the 52d experiment of his last series, our author concludes—

"That *bile* accelerates the solution of oil, by the gastric juice; and I have no doubt, it facilitates the chymification of all fatty and oily aliments; and is required, and necessarily called into the stomach *only* for that purpose. This has been frequently indicated in the course of these experiments, by the effect which it has produced on fatty or oily aliments, when adventitiously mixed with gastric juice."—p. 264.

The following are the general and particular inferences which our author makes from all his experiments:

*Inferences, from the foregoing Experiments and Observations.*

1. "That *animal* and *farinaceous* aliments are more easy of digestion than *culinary vegetable.*

2. That the susceptibility of digestion does not, however, depend altogether upon *natural* or *chemical* distinctions.

3. That digestion is facilitated by *minuteness* of *division* and *tenderness* of *fibre,* and retarded by opposite qualities.

4. That the *ultimate principles* of aliment are always the same, from whatever food they may be obtained.

5. That the action of the stomach, and its fluids are the same on *all kinds* of diet.

6. That the *digestibility* of aliment does not depend upon the *quantity* of nutrient principles that it contains.

7. That the *quantity* of food generally taken, is more than the wants of the system require; and that such excess, if persevered in, generally produces, not only functional aberration, but disease of the coats of the stomach.

8. That *bulk,* as well as *nutriment,* is necessary to the articles of diet.

9. That *oily* food is difficult of digestion, though it contains a large proportion of nutrient principles.

10. That the *time* required for the digestion of food, is various, depending upon the quantity and quality of the food, of the stomach, &c.; but that the time ordinarily required for the disposal of a moderate meal of the fibrous parts of meat, with bread, &c., is from three to three and a half hours.

11. That *solid* food, of a certain texture, is easier of digestion, than *fluid.*

12. That stimulating *condiments* are injurious to the healthy stomach.

13. That the use of *ardent spirits always* produces disease of the stomach, if persevered in.

14. That *hunger* is the effect of *distention* of the vessels that secrete the gastric juice.

15. That the processes of *mastication, insalivation* and *deglutition,* in an abstract point of view, do not, in any way, affect the digestion of food; or, other words, when food is introduced directly into the stomach, in a finely divided state, without these previous steps, it is as readily and as perfectly digested as when they have been taken.

16. That *saliva* does not possess the properties of an alimentary solvent.

17. That the *first* stage of digestion is effected in the stomach.

18. That the natural *temperature* of the stomach is 100 deg. Fahrenheit.

19. That the temperature is *not elevated* by the indigestion of food.

20. That *exercise elevates* the temperature; and that *sleep* or *rest,* in a recumbent position, *depresses* it.

21. That the *agent* of chymification is the *Gastric Juice.*

22. That it acts as a *solvent* of food, and alters its properties.

23. That its action is facilitated by the *warmth* and *motions* of the stomach.

24. That it contains free *Muriatic Acid* and some other *chemical* principles.

25. That it is never found *free* in the gastric cavity; but is always excited to discharge itself by the introduction of *food,* or other irritants.

26. That it is secreted from vessels distinct from the mucous follicles.

27. That it is seldom obtained pure, but is generally mixed with mucus, and sometimes with saliva. When pure, it is capable of being kept for months, and perhaps for years.*

28. That it *coagulates* albumen, and afterwards *dissolves coagulæ.*

29. That it *checks* the progress of putrefaction.

30. That the pure gastric juice is fluid, *clear* and *transparent;* without *odour;* a little *salt,* and perceptibly *acid.*

31. That like other chemical agents, it *commences* its action on food, as soon as it comes in *contact* with it.

32. That it is capable of *combining* with a certain and fixed *quantity* of food, and when more aliment is presented for its action than it will dissolve, disturbance of the stomach, or "indigestion," will ensue.

*I have now (Nov. 1, 1833) in my possession, some clear gastric juice, possessing all its original properties, unchanged and undiminished, which was taken from the stomach in Dec. 1832, about eleven months ago, and has been kept tightly corked in vials.

33. That it becomes intimately *mixed* and *blended* with the ingestæ in the stomach, by the motions of that organ.

34. That it is *invariably* the *same substance,* modified only by *admixture* with other fluids.

35. That *gentle exercise* facilitates the digestion of food.

36. That *bile* is not ordinarily found *in the stomach,* and is *not* commonly *necessary* for the digestion of food; but

37. That, when *oily* food has been used, it assists its digestion.

38. That *chyme* is *homogeneous,* but variable in its *colour* and *consistence.*

39. That towards the *latter stages* of chymification, it becomes more *acid* and *stimulating,* and passes more rapidly from the stomach.

40. That *water, ardent spirits,* and most other *fluids* are not affected by the gastric juice, but pass from the stomach soon after they have been received.

41. That the *inner coat* of the stomach, is of a pale *pink* colour, varying in its hues, according to its full or empty state.

42. That, in health, it is constantly sheathed with a *mucous* coat.

43. That the gastric juice and mucus are *dissimilar* in their *physical* and *chemical* properties.

44. That the appearance of the interior of the stomach, *in disease,* is essentially different from that of its *healthy* state.

45. That the motions of the stomach produce a constant *churning* of its contents, and *admixture* of food and gastric juice.

46. That these motions are in two directions; *transversely* and *longitudinally.*

47. That the *expulsion* of the chyme is assisted by a *transverse band, &c.*

48. That *chyle* is formed in the duodenum and small intestines, by the action of *bile* and *pancreatic juice,* on the chyme.

49. That crude *chyle* is a *semi-transparent, whey-coloured* fluid.

50. That it is further changed by the action of the *lacteals* and *mesenteric glands.* This is only an *inference* from the other facts. It has not been the subject of experiment.

51. That *no other* fluid produces the same effect on food that gastric juice does; and that it is the *only solvent* of *aliment.*"—pp. 275–78.

We have thus given an ample account of Dr. Beaumont's book, or rather such specimens of it, as will put our readers in possession of the most interesting results which it offers. We hope that all who may find it within their reach, will buy and read it for themselves. Since the days of Spallanzani, the experiments of Dr. Beaumont are the most important that have been made on the subject of digestion, and they go fully, we think, to establish the doctrine of digestion by a solvent, secreted by the stomach when stimulated by food.

